# Evaluation of Recombinant Attenuated *Salmonella* Vaccine Strains for Broad Protection against Extraintestinal Pathogenic *Escherichia coli*

**DOI:** 10.3389/fimmu.2017.01280

**Published:** 2017-10-09

**Authors:** Jacob T. Maddux, Zachary R. Stromberg, Roy Curtiss III, Melha Mellata

**Affiliations:** ^1^The Biodesign Institute, Arizona State University, Tempe, AZ, United States; ^2^Department of Food Science and Human Nutrition, Iowa State University, Ames, IA, United States; ^3^School of Life Sciences, Arizona State University, Tempe, AZ, United States

**Keywords:** vaccine, extraintestinal pathogenic *Escherichia coli*, *Salmonella*, sepsis, urinary tract infection

## Abstract

Antibiotic-resistant bacterial infections are difficult to treat, producing a burden on healthcare and the economy. Extraintestinal pathogenic *Escherichia coli* (ExPEC) strains frequently carry antibiotic resistance genes, cause infections outside of the intestine, and are causative agents of hospital-acquired infections. Developing a prevention strategy against this pathogen is challenging due to its antibiotic resistance and antigenic diversity. *E. coli* common pilus (ECP) is frequently found in ExPEC strains and may serve as a common antigen to induce protection against several ExPEC serotypes. In addition, live recombinant attenuated *Salmonella* vaccine (RASV) strains have been used to prevent *Salmonella* infection and can also be modified to deliver foreign antigens. Thus, the objective of this study was to design a RASV to produce ECP on its surface and assess its ability to provide protection against ExPEC infections. To constitutively display ECP in a RASV strain, we genetically engineered a vector (pYA4428) containing aspartate-β-semialdehyde dehydrogenase and *E. coli ecp* genes and introduced it into RASV χ9558. RASV χ9558 containing an empty vector (pYA3337) was used as a control to assess protection conferred by the RASV strain without ECP. We assessed vaccine efficacy in *in vitro* bacterial inhibition assays and mouse models of ExPEC-associated human infections. We found that RASV χ9558(pYA4428) synthesized the major pilin (EcpA) and tip pilus adhesin (EcpD) on the bacterial surface. Mice orally vaccinated with RASV χ9558(pYA3337) without ECP or χ9558(pYA4428) with ECP, produced anti-*Salmonella* LPS and anti-*E. coli* EcpA and EcpD IgG and IgA antibodies. RASV strains showed protective potential against some *E. coli* and *Salmonella* strains as assessed using *in vitro* assays. In mouse sepsis and urinary tract infection challenge models, both vaccines had significant protection in some internal organs. Overall, this work showed that RASVs can elicit an immune response to *E*. *coli* and *Salmonella* antigens in some mice, provide significant protection in some internal organs during ExPEC challenge, and thus this study is a promising initial step toward developing a vaccine for prevention of ExPEC infections. Future studies should optimize the ExPEC antigens displayed by the RASV strain for a more robust immune response and enhanced protection against ExPEC infection.

## Introduction

Extraintestinal pathogenic *Escherichia coli* (ExPEC) is a heterogeneous group of bacteria that causes extraintestinal diseases in humans and costs the US healthcare system over $1 billion annually (1). Human ExPEC strains can be subclassified into neonatal meningitis-causing *E. coli*, sepsis-associated *E. coli*, and uropathogenic *E. coli* that causes urinary tract infections (UTIs). In the US, ExPEC infections account for 17% of severe sepsis cases, are the primary cause of community-acquired UTI, and cause ~50% of nosocomial UTI (2, 3). Antibiotic-resistant ExPEC strains complicate treatment of these infections (4), but vaccination as an alternative to antibiotic treatment or a combined strategy against ExPEC may have a significant benefit to public health (5–7). Currently, no licensed vaccine exists for prevention of ExPEC in humans and those developed have lacked immunogenicity, safety, and cross-protectiveness (5).

Effective vaccine targets should be broadly protective against several ExPEC serotypes. *E. coli* common pilus (ECP) is an extracellular adhesin frequently present in *E. coli* and some other *Enterobacteriaceae*, e.g., *Enterobacter cancerogenus, Klebsiella pneumoniae*, and *Serratia odorifera* (8, 9). The *ecpRABCDE* operon encodes for a transcriptional regulator (EcpR), a major Pilin (EcpA), a putative chaperone (EcpB), an usher (EcpC), a tip pilus adhesin (EcpD), and a potential chaperone (EcpE) (8). In *E. coli*, ECP promotes biofilm formation on inert surfaces and contributes to colonization of human epithelial cell lines *in vitro* (8–13). Mutant *ecp* ExPEC strains have reduced ability to invade *ex vivo* mouse bladders (13). Virulence was also reduced in an avian pathogenic *E. coli* mutant *ecp* strain that had decreased ability to cause sepsis in chickens (10). Vaccination with ECP recombinant antigens was protective in a lethal mouse sepsis model (14). Additionally, ECP was produced in *E. coli* in urine samples from patients with UTI (13). These studies lend evidence that ECP may be a good vaccine antigen.

A vaccine that could provide protection against multiple pathogens would be highly desired considering its potential wide use and economic benefits. Antigen delivery by recombinant attenuated *Salmonella* vaccine (RASV) strains has been used to induce immune responses against both the carrier *Salmonella* and foreign protective antigens from bacteria, viruses, and protozoa (15–20). RASVs can be delivered orally, which eliminates use of needles and syringes, and thus is an affordable choice for mass vaccination. Recently developed RASVs have the advantage of multiplying like wild-type organisms in the early phase of colonization and become avirulent following invasion into internal organs (15, 20). The objectives of this study were to (i) genetically engineer a RASV strain to synthesize and display *E. coli* EcpA and EcpD antigens; (ii) assess the ability of the RASV to elicit serum and mucosal immune responses in mice; (iii) evaluate the protective potential of the RASV against ExPEC and *Salmonella* using *in vitro* assays; and (iv) assess the RASV protective ability in animal models of ExPEC-associated human sepsis and UTI.

## Materials and Methods

### Ethics Statement

This study was carried out in accordance with the recommendations of Arizona State University Institutional Animal Care and Use Committee. The protocol (#1168R) was approved by the Arizona State University Institutional Animal Care and Use Committee. Six-week-old female BALB/c mice (Charles River Laboratories, Wilmington, MA, USA) and 4-week-old female CBA/J mice (Jackson Laboratories, Bar Harbor, ME, USA) were obtained for infection experiments. Mice were acclimated for 7 days before experiments began. During the experiments, animals were monitored twice daily by our team, animal caretakers, and further inspected by a veterinarian.

### Bacterial Strains, Plasmids, and Growth Conditions

Strains and plasmids used are listed in Table 1. Strains were stored as stock cultures at −80°C in peptone-glycerol medium. Unless otherwise specified, strains were grown in lysogeny broth (LB) containing 0.1% glucose. *Salmonella enterica* serovar Typhimurium attenuated strain χ9558, derived from the virulent *S*. Typhimurium strain UK-1 (χ3761), using strategies that enhance safety and immunogenicity (21, 22), was used to deliver ECP. Urosepsis strain CFT073 (23) was used in animal challenge experiments. ExPEC strains CFT073, JJ1886, UTI89, and RS218, non-pathogenic *E. coli* strains HS-4, J198, and Nissle 1917, laboratory *E. coli* MG1655, and *S*. Typhi χ3444, *S*. Typhimurium χ3761, and *S*. Paratyphi χ8387 strains were used for *in vitro* assays.

**Table 1:** Bacterial strains and plasmids.

Strain or plasmid	Relevant genotype, phenotype, and characteristics	Reference

*Salmonella enterica*		
χ3444	*Salmonella* Typhi	This study
χ3761	*Salmonella* Typhimurium UK-1	(24)
χ8387	*Salmonella* Paratyphi	(25)
χ9558	Δ*pmi-2426* Δ(*gmd-fcl*)*-26* ΔP_fur81_:TT *araC* P_BAD_ *fur* ΔP_crp527_:TT *araC* P^_BAD_^ *crp* Δ*asdA27*:TT *araC* P_BAD_ *c2* Δ*araE25* Δ*araBAD23* Δ*relA198*:*araC* P^_BAD_^ *lacI* TT Δ*sopB1925* Δ*agfBAC811*	(21)

***Escherichia coli***		

χ6212	Δ*asdA* DH5α derivative	(26)
E24377A	Human enterotoxigenic *E. coli*	(27)
CFT073	UPEC, O6:K2:H1, ST73, acute pyelonephritis	(23)
JJ1886	UPEC, O25b:H5, ST131, fatal urosepsis	(28)
UTI89	UPEC, O18:K1:H7, ST95, Cystitis	(29)
RS218	NMEC, O18:K1:H7, ST95	(30)
HS-4	Human non-pathogenic commensal, O9:H4	(29)
J198	Human non-pathogenic commensal, O22	(31)
Nissle 1917	Human non-pathogenic commensal, O6:K5:H1	(32)
MG1655	Laboratory strain of *E. coli* K-12, OR:K-:H48	(29)

**Plasmid**		

pCR-XL-TOPO	3.5 kb pUC *ori* cloning vector, Kanamycin resistance	Invitrogen
pYA3337	*asd*-based cloning vector (pSC101 *ori*) with P*_trc_* promoter	(33)
pYA4428	The *ecpABCD* was cloned under P_trc_ of pYA3337	This study

*NMEC, neonatal meningitis E. coli; ST, sequence type; UPEC, uropathogenic E. coli*.

Recombinant attenuated *Salmonella* vaccine strains and challenge strain CFT073 were grown statically overnight in LB. The next day, the culture was inoculated 1:100 into fresh LB. RASV strains were grown with aeration at 37°C to OD_600_ of ~0.85. CFT073 was grown with aeration at 37°C to OD_600_ of ~0.85 (sepsis challenge) or statically overnight (intraurethral challenge). Strains were harvested by centrifugation at 24°C and resuspended in PBS.

### Construction of *asd*-Positive *ecp* Plasmid Vaccine Vector

Fragment *ecpABCD*, a 5622 bp portion of the *ecp* operon comprising *ecpRABCDE*, was PCR-amplified from genomic DNA of *E. coli* strain E24377A using KlenTaq LA DNA Polymerase (DNA Polymerase Technology, Inc., Saint Louis, MO, USA) and primer sets: P1: *ecpA* (BsrGI)-F: 5′ TAGTAATGTAC****A**TG**AAAAAAAAGGTTCTGGCAATAG 3′ and P2: *ecpD* (HindIII)-R: 5′ CCCAAGCTTGGG**TTA**GTTAATGTTACGCCACCGTCGCC 3′ (Figure S1 in Supplementary Material). The amplified fragment included the 5′ region of the first gene, *ecpA*, from its start codon through the stop codon of the last gene, *ecpD*. To introduce the enzyme recognition site for BsrGI into vector pYA3337, we amplified the vector plasmid using primer sets: P3: pYA3337 (HindIII) F 5′ CCACAAGCTTGGCTGTTTTGGCGGATGAGA 3′ and P4: pYA3337 (BsrGI) R 5′ CCTATGTACATGTTTCCTGTGTGAAATTG 3′. The amplified fragment included at the 5′ region, the enzyme restriction site HindIII, and at the 3′ region, the enzyme restriction site BsrGI. PCR products were cloned into the pCR-XL-TOPO vector according to the manufacturer’s instructions (Invitrogen).

PCR products of *asd*-positive vector pYA3337 (BsrG1-positive) and *ecpABCD* fragment cloned into the pCR-XL-TOPO were cut using HindIII and BsrGI enzymes, respectively. DNA bands of the digested *ecpABCD*_(HindIII, BsrGI)_ and pYA3337_(HindIII, BsrGI)_ were purified from an agarose gel and ligated together using T4 DNA ligase (New England Biolabs, Ipswich, MA, USA) to generate plasmid pYA4428. Plasmids were verified by PCR, on agarose gel, restriction digestion analysis with HindIII and BsrGI, and sequencing.

The recombinant plasmid was first introduced into *E. coli* strain χ6212 commonly used for synthesis of foreign proteins (26). The purified plasmid obtained from χ6212 was then electroporated into competent cells of *asd*-negative *Salmonella* vaccine strain χ9558 to obtain the balanced-lethal construct. Selection for transformants was achieved by growth on LB agar plates and verified by PCR amplification.

### Evaluation of ECP Synthesis

Bacterial ECP synthesis was evaluated by sodium dodecyl sulfate-polyacrylamide gel electrophoresis (SDS-PAGE) and Western blot with rabbit anti-EcpA and -EcpD antibodies. The *E*. *coli* strain E24377A was used as a positive control and χ9558 containing *ecpABC, ecpABCD*, or *ecpRABCDE* were tested to determine which portion of the operon maintains ECP synthesis. χ9558 containing *ecpABC* or *ecpRABCDE* were constructed using the aforementioned methods. Surface display of ECP was visualized by transmission electron microscopy (TEM) using rabbit anti-EcpA and -EcpD antibodies and goat anti-rabbit IgG with 10 nm colloidal gold (MP Biomedicals, Santa Ana, CA, USA) at a concentration of 1:250 (9).

### Vaccination and Antibody Responses

Mice were orally (behind the incisors) administered 20 µl using a pipette containing either PBS (unvaccinated), 10^9^ CFU of χ9558(pYA3337) that carries the *asd*-plasmid with no *ecp* genes, or 10^9^ CFU χ9558(pYA4428) that carries the *asd*-plasmid with *ecpABCD* (Figure S2 in Supplementary Material). Before immunization, mice were deprived of food and water for 4 h and resupplied 30 min after vaccination. Serum was obtained at days 20 and 41 (BALB/c mice) and days 20 and 30 (CBA/J mice) post-immunization from blood collected from the submandibular vein. Vaginal wash samples were obtained at days 28 and 41 (BALB/c mice) and day 28 (CBA/J mice) by repeated flushing of the vaginal tract and aspiration of 50 µl of PBS. Serum IgG and vaginal IgA and IgG responses against ECP proteins and *Salmonella* LPS were determined by ELISA as described previously (14). Briefly, *E*. *coli* antigens were PCR amplified and cloned into pET-101/D-TOPO vectors (Invitrogen) and expressed in *E*. *coli* strain BL21 as His-tagged proteins. Proteins were purified using ProBond Ni-NTA resin columns (Invitrogen). Endotoxin removal spin columns (Pierce Biotechnology, Rockford, IL, USA) were used to remove any remaining LPS from purified proteins. Commercial *S*. Typhimurium LPS (Sigma) was used as a source of LPS. Plates were coated at a concentration of 2.0 µg/ml of antigen and incubated overnight at 4°C. The remaining steps were performed at room temperature. Plates were washed with PBS containing 0.05% Tween-20 and blocked for 1 h with SEA BLOCK (Thermo Scientific). Serum or vaginal wash samples were added at 1:50 or 1:10, respectively. Samples were diluted twofold down the plate, incubated for 1 h, and washed. Goat anti-mouse IgG for serum and day 41 vaginal wash samples from BALB/c mice (1:5,000; Southern Biotech, Birmingham, AL, USA) or goat anti-mouse IgA for all vaginal wash samples (1:5,000; Southern Biotech) was added and plates were incubated for 1 h. Plates were washed, and streptavidin, alkaline phosphatase conjugate (1:2,000; Southern Biotech) was added and incubated for 1 h. After incubation and washing, p-nitrophenyl phosphate (Thermo Scientific) was added according to the manufacturer’s protocol. The reaction was stopped with 2 N NaOH and plates were read at 405 nm. The endpoint titer was set as the reciprocal of the highest dilution that gave an OD_405_ twice that of the unvaccinated control.

Antibodies elicited against ECP were further evaluated by Western blot. Serum collected on day 41 was pooled in equal amounts from 10 mice per group. From unvaccinated, χ9558(pYA3337) and χ9558(pYA4428) immunized BALB/c mice pooled serum was used as a substitute for rabbit anti-EcpA to probe against purified EcpA by Western blot as described above.

### Serum and Vaginal Wash Bacterial Inhibition Assays

Bacterial inhibition was tested using bacterial strains (Table 1) in pooled (*n* = 8/group, in equal volumes) serum or vaginal wash samples obtained on day 41 from BALB/c mice. Bacterial colonies from a fresh LB agar plate were suspended in M9 minimal media until OD_600_ reached 0.1. The suspension was diluted in M9 media (1 × 10^2^ CFU), mixed with an equal volume of pooled serum or vaginal wash samples, and incubated at 37°C for 6 h. After incubation, the mixture was serially diluted and plated on MacConkey agar to determine viable counts. Samples were tested in duplicate in two independent experiments.

### Protection Studies

Mouse models of human sepsis and UTI were used to evaluate the protective ability of RASV immunization. For the mouse sepsis model, BALB/c mice were intraperitoneally challenged with 100 µl of PBS containing 10^8^ CFU of CFT073 on day 41 postvaccination. Previously, 100% lethality was observed in a mouse sepsis model using 10^8^ CFU of CFT073 by 7 days postchallenge (34). In order to quantitatively determine bacterial loads in internal organs, previous studies have used early endpoints of 24–48 h postchallenge with CFT073 (35, 36). Therefore, mice were euthanized before lethality at 24 h postchallenge, and blood, liver, and spleen were collected, serially diluted, and plated on MacConkey agar for enumeration of *E. coli*.

For the mouse UTI model, CBA/J mice were intraperitoneally anesthetized with a cocktail of ketamine (100 mg/kg), xylazine (10 mg/kg), and acepromazine (2.5 mg/kg) and the bladder was emptied by gentle pressure on the abdomen. The median infection dose for transurethral inoculation of CFT073 was determined previously as 10^6^ CFU/mouse (35). To ensure infection, mice were inoculated with 50 µl of PBS containing 10^8^ CFU of CFT073 *via* transurethral catheterization using a sterile polyethylene catheter (Intramedic, Becton Dickinson, Sparks, MD, USA) on day 28 postvaccination. At 48 h postchallenge, mice were euthanized and bladder, kidney, liver, and spleen samples were collected, serially diluted, and plated on MacConkey agar for enumeration of *E. coli*.

### Statistical Analysis

An ANOVA followed by Tukey’s test for multiple comparisons was used to compare between groups for ELISAs, *in vitro* sera and vaginal wash assays, and the sepsis and UTI models. Fisher’s exact test (two-tailed) was used to compare treatments for the proportion of tissues positive for *E. coli* in the UTI mouse model. Analyses were carried out in GraphPad Prism 6.0. *P* values <0.05 were considered significant.

## Results

### ECP Synthesis in RASV Strain χ9558(pYA4428)

As screened on the SDS-PAGE gel stained with Coomassie blue and Western blot (Figure S3 in Supplementary Material), ECP production was detected in χ9558(pYA4428) but not in χ9558(pYA3337). EcpA and EcpD were displayed on the surface of χ9558(pYA4428) as shown by TEM (Figure S4 in Supplementary Material).

### Level of Antigen-Specific Antibodies Elicited in BALB/c and CBA/J Mice

**Figure 1:**
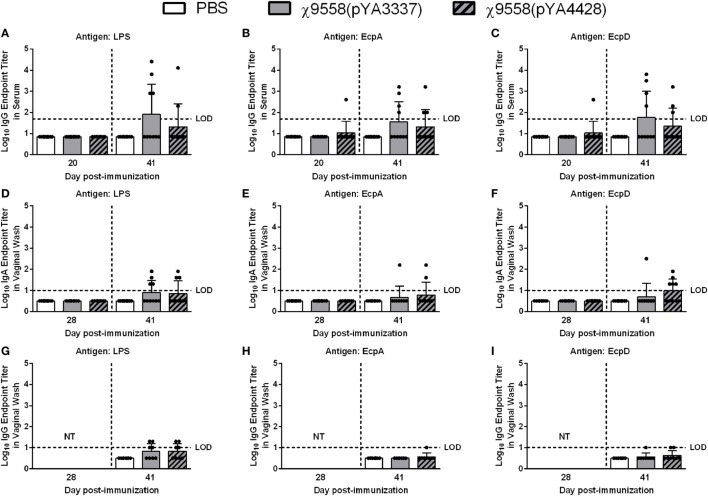
Antibody responses against *Salmonella* LPS and *Escherichia coli* common pilus antigens in vaccinated BALB/c mice. Data represent **(A–C)** serum IgG **(D–F)** vaginal wash IgA, and **(G–I)** vaginal wash IgG antibody levels induced in BALB/c mice immunized with PBS, χ9558(pYA3337), or χ9558(pYA4428). Individual samples from 8 to 10 mice per treatment were analyzed by ELISA against *Salmonella* LPS and *E. coli* EcpA and EcpD. Day 28 vaginal wash samples were not tested (NT) for IgG. Bars represent the mean and each dot represents an individual mouse. The horizontal dashed line represents the limit of detection (LOD). Samples that tested negative were assigned a value halfway between zero and the LOD. Error bars represent SDs.

To assess immune responses to vaccination, serum and vaginal wash samples from 8 to 10 individual mice per treatment were evaluated by ELISA for anti-*Salmonella* LPS and anti-*E. coli* (EcpA and EcpD) antibodies. For BALB/c mice on day 20, only one mouse vaccinated with χ9558(pYA4428) elicited anti-EcpA and EcpD IgG antibodies, and no IgG antibodies were observed for LPS (Figure 1). On day 28, no IgA antibodies were detected in vaginal wash samples or if elicited were below the limit of detection in the ELISA. On day 41, some mice vaccinated with χ9558(pYA3337) or χ9558(pYA4428) elicited IgG and IgA antibodies to all three antigens, except that no IgG antibodies were detected against EcpA from vaginal wash samples of χ9558(pYA3337) immunized mice. Although some RASV immunized mice had elevated antibody titers compared with unvaccinated mice, no significant differences were observed between vaccinated and unvaccinated mice or between vaccination groups.

**Figure 2:**
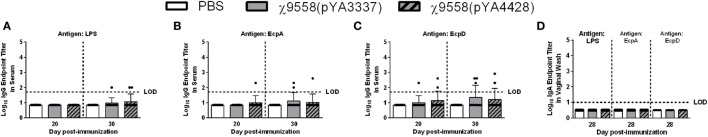
Antibody responses against *Salmonella* LPS and *Escherichia coli* common pilus antigens in vaccinated CBA/J mice. Data represent **(A–C)** serum IgG and **(D)** vaginal wash IgA antibody levels induced in CBA/J mice immunized with PBS, χ9558(pYA3337), or χ9558(pYA4428). Individual samples from 10 mice per treatment were analyzed by ELISA against *Salmonella* LPS and *E*. *coli* EcpA and EcpD. Bars represent the mean and each dot represents an individual mouse. The horizontal dashed line represents the limit of detection (LOD). Samples that tested negative were assigned a value halfway between zero and the LOD. Error bars represent SDs.

For CBA/J mice on day 20, serum and vaginal wash samples from ten individual mice per group were tested by ELISA. Only one χ9558(pYA4428) vaccinated mouse but not χ9558(pYA3337), elicited anti-EcpA IgG antibodies, one χ9558(pYA3337) and two χ9558(pYA4428) immunized mice elicited anti-EcpD IgG antibodies, and no anti-LPS IgG antibodies were observed (Figure 2). Similar to unvaccinated mice, anti-LPS, anti-EcpA, and anti-EcpD IgA antibodies were not detected in vaginal washes of vaccinated mice on day 28. On day 30, one χ9558(pYA3337) and two χ9558(pYA4428) immunized mice elicited anti-LPS IgG antibodies, two χ9558(pYA3337) and one χ9558(pYA4428) immunized mice elicited anti-EcpA IgG antibodies, and three χ9558(pYA3337) and two χ9558(pYA4428) immunized mice elicited anti-EcpD IgG antibodies.

To determine whether antibodies against ECP were also detected by Western blot, pooled serum collected from BALB/c mice (*n* = 10/group) was used to probe purified EcpA. Western blot analysis showed no reaction to purified EcpA when probed with serum extracted from unvaccinated or χ9558(pYA3337) immunized mice (Figure S5 in Supplementary Material). A positive reaction was observed for serum from χ9558(pYA4428) immunized mice. Thus, we confirmed our ELISA results for χ9558(pYA4428) but could not confirm our results for χ9558(pYA3337) by Western blot.

### Inhibitory Effect of Mouse Sera and Vaginal Wash Samples on Bacterial Strains

**Figure 3:**
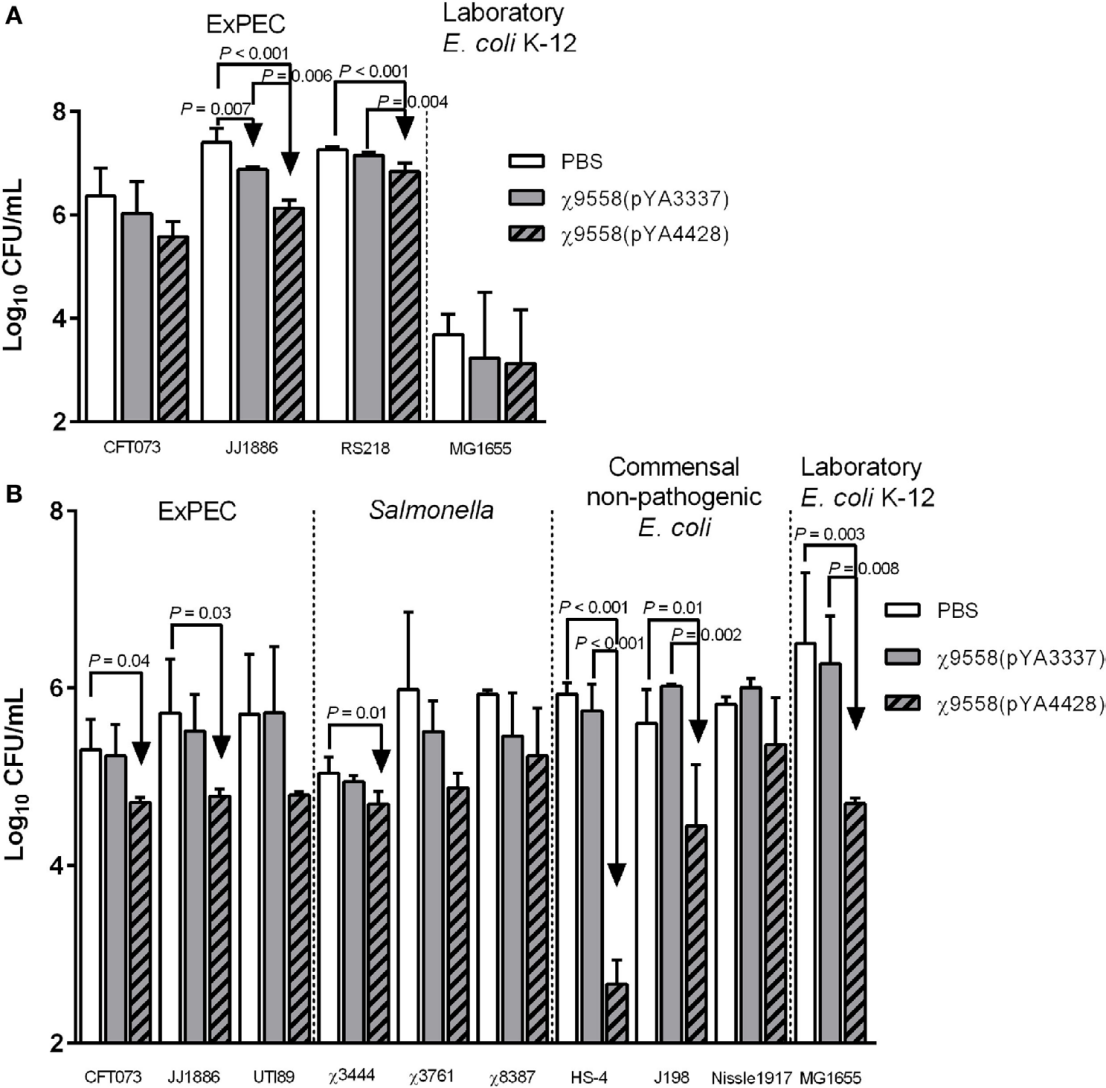
Bacterial inhibition of serum and vaginal wash samples from BALB/c mice. **(A)** Serum or **(B)** vaginal wash samples were mixed 1:1 with bacterial strain cultures and incubated at 37°C for 6 h. After incubation, mixtures were plated on MacConkey agar for bacterial enumerations. Bacterial levels between groups were compared by an ANOVA followed by Tukey’s method for multiple comparisons. *P* values <0.05 were considered significant. Error bars represent SDs.

Pooled mouse serum and vaginal wash samples from 10 mice by group were mixed with bacterial strains and incubated for 6 h to determine whether antibodies or other antimicrobial products elicited from RASV immunization influenced bacterial levels. These assays were used to test inhibitory activity of serum and vaginal wash samples against multiple pathogenic bacterial strains including ExPEC and *Salmonella* strains not tested *in vivo* and non-pathogenic *E*. *coli* strains that represent organisms inherent in the gastrointestinal tract of humans. A limited amount of sera was collected during the study, and therefore, heat-inactivation of sera was not used as a control. Generally, sera from mice vaccinated with χ9558(pYA4428) had the strongest inhibition against strains (Figure 3). The urosepsis strain JJ1886 was significantly decreased in serum samples of both χ9558(pYA3337) and χ9558(pYA4428) vaccinated mice compared with unvaccinated mice (Figure 3A). A significant decrease was also observed in serum of χ9558(pYA4428) compared with χ9558(pYA3337) immunized mice. The neonatal meningitis-causing *E. coli* strain RS218 was significantly decreased in serum from χ9558(pYA4428) immunized mice but not χ9558(pYA3337) compared with unvaccinated mice. A significant decrease in levels of RS218 was detected in serum of χ9558(pYA4428) compared with that of χ9558(pYA3337) immunized mice. No significant differences were observed for urosepsis strain CFT073 or laboratory *E. coli* strain MG1655 between treatments.

Cervicovaginal lavage samples from healthy women have been shown to inhibit growth of *E. coli ex vivo* (37). To determine if RASV immunization influences bacterial inhibition, various ExPEC, *Salmonella*, and non-pathogenic *E. coli* strains were tested for growth in vaginal wash samples (Figure 3B). Two of three ExPEC strains (CFT073 and JJ1886) were significantly reduced in vaginal washes from χ9558(pYA4428) immunized mice but not χ9558(pYA3337) compared to unvaccinated mice. One of three *Salmonella* strains (*S*. Typhi χ3444), two of three commensal non-pathogenic *E. coli* strains (HS-4 and J198), and laboratory *E. coli* strain MG1655 had significantly decreased levels in vaginal washes from χ9558(pYA4428) vaccinated mice but not in that of χ9558(pYA3337) compared to unvaccinated mice. No significant differences were detected between χ9558(pYA3337) vaccinated and unvaccinated mice or between any of the treatments for ExPEC strain UTI89, *S*. Typhimurium χ3761, *S*. Paratyphi χ8387, and *E. coli* Nissle 1917.

### Vaccine Protection against CFT073 Challenge in Mice

**Figure 4:**
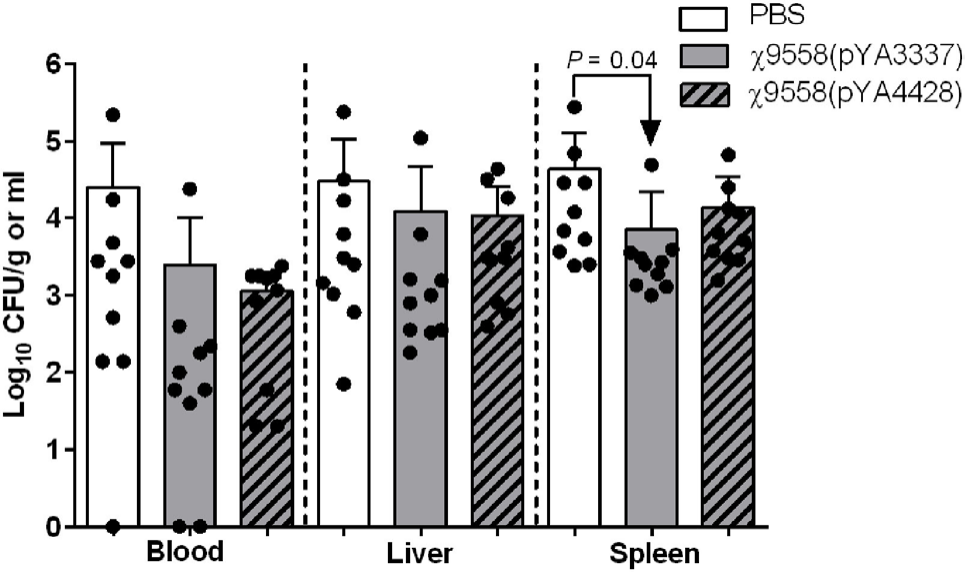
Effect of vaccination on extraintestinal pathogenic *Escherichia coli* strain CFT073 ability to cause sepsis in mice. Female BALB/c mice were immunized with PBS, χ9558(pYA3337), or χ9558(pYA4428), challenged intraperitoneally with 10^8^ CFU of CFT073, and assessed 24 h postchallenge for bacterial concentration in the blood, liver, and spleen. Each experimental group contained 10 mice. Each dot represents an individual mouse and vertical dashed lines separate sample type. Bacterial loads of mice between groups were compared by an ANOVA followed by Tukey’s method for multiple comparisons. *P* values <0.05 were considered significant. Error bars represent SDs.

After vaccination, all mice survived and no clinical signs of disease due to *Salmonella* infection (diarrhea, weight loss, etc.) were observed. In the mouse model of sepsis, BALB/c mice were intraperitoneally challenged with 10^8^ CFU of CFT073 to assess the efficacy of vaccine treatment against systemic infection. BALB/c mice were selected based on past sepsis studies using this genetic background to evaluate ExPEC vaccines (36, 38). The only significant difference relating to bacterial loads in the mouse sepsis model was in the spleen of mice vaccinated with χ9558(pYA3337) (Figure 4).

**Figure 5:**
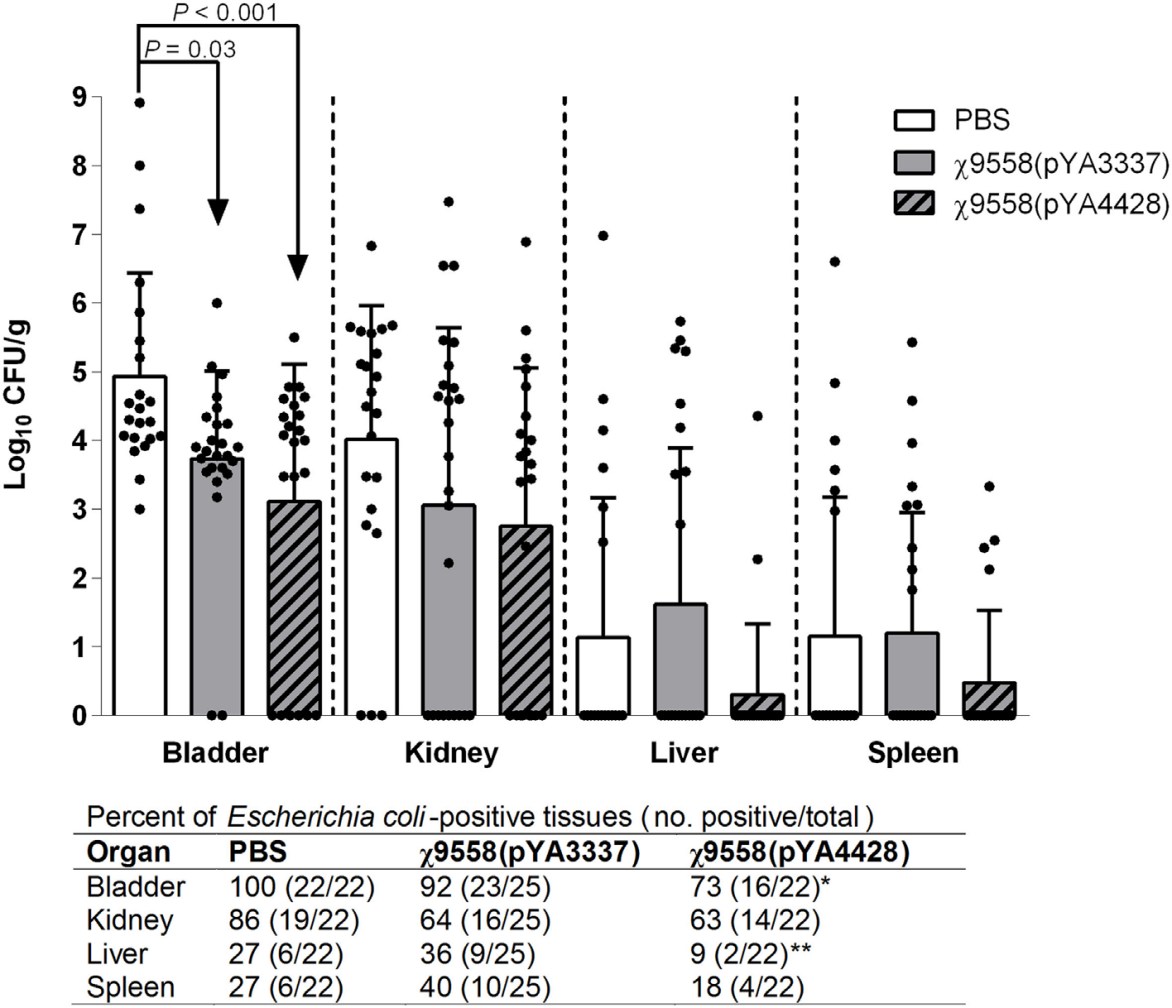
Effect of vaccination on extraintestinal pathogenic *Escherichia coli* strain CFT073 ability to cause urinary tract infection in mice. Female CBA/J mice were immunized with PBS, χ9558(pYA3337), or χ9558(pYA4428), challenged intraurethrally with 10^8^ CFU of CFT073, and assessed 48 h postchallenge for bacterial concentration in the bladder, kidney, liver, and spleen. Each experimental group contained at least 22 mice. Each dot represents an individual mouse and vertical dashed lines separate tissue type. Bacterial loads between groups were compared by an ANOVA followed by Tukey’s method for multiple comparisons. Below the *x*-axis for the number of *E. coli*-positive tissues, an asterisk (*) represents a significant (*P* < 0.05) difference for a vaccine treatment compared with the unvaccinated treatment, and two asterisks (**) represents a significant (*P* < 0.05) difference for χ9558(pYA4428) compared with χ9558(pYA3337) immunization as determined by Fisher’s exact test. Error bars represent SDs.

In the UTI mouse model, CBA/J mice were intraurethrally challenged with 10^8^ CFU of CFT073 to determine the impact of vaccination on bacterial loads in urinary system organs (bladder and kidney) and other internal organs (liver and spleen) (Figure 5). CBA/J mice were selected based on an established UTI protocol (39) to assess the efficacy of vaccine treatment against UTI, and to assess whether a different genetic background resulted in a response similar to RASV immunization of BALB/c mice. In general, mice vaccinated with RASV strains had numerically lower bacterial loads in organs. Significantly lower bacterial loads than unvaccinated mice were observed in the bladder for both χ9558(pYA3337) and χ9558(pYA4428). Also, χ9558(pYA4428) but not χ9558(pYA3337) vaccinated mice had significantly fewer number of *E. coli*-positive bladder samples than unvaccinated mice. In the kidney, liver, and spleen, bacterial loads for both χ9558(pYA3337) and χ9558(pYA4428) were not significantly different than unvaccinated mice. However, for the liver, χ9558(pYA4428) vaccinated mice had significantly fewer number of *E. coli*-positive samples than χ9558(pYA3337), but was not significantly different from unvaccinated mice.

## Discussion

Recombinant attenuated *Salmonella* vaccine strains colonize the intestinal mucosa and other lymphoid tissues to elicit mucosal and systemic immunity, which is important for protection against an invasive pathogen like ExPEC (40). Previously, RASV χ9558 delivering a pneumococcal antigen replicated and colonized the Peyer’s patches, spleen, and liver of mice for at least 3 weeks post-vaccination (41). Also, as used in the current study, RASV strains with a regulated delayed lysis *in vivo* system colonize the spleen at levels around 10^5^ CFU 1-week postimmunization (15). After RASV immunization, sufficient time and successful display of the antigen is needed for the development of a humoral response. Other studies have used type II and III secretion systems for RASV surface display of foreign antigens (41–44). Here, by cloning *ecpABCD* genes into the *asd*-based low copy vector pYA3337, *E. coli* chaperone EcpB and usher EcpC successfully displayed EcpA and EcpD on the surface of RASV χ9558(pYA4428). Using one plasmid to synthesize two different antigens and displaying them on the RASV surface is unique and could be used with other antigens for applications in biotechnology, microbiology, and vaccinology.

An immune response is a key component of an effective vaccine. As expected, vaccination with χ9558(pYA4428) elicited both anti-*Salmonella* LPS and anti-*E. coli* ECP antibodies in mice. Anti-EcpA antibodies were detected in mice vaccinated with χ9558(pYA4428) earlier than other treatments, which suggests inclusion of ECP in the RASV may elicit a faster immune response to some *E. coli* antigens. However, the ability of χ9558(pYA3337) to elicit anti-EcpA and -EcpD antibodies indicates cross-immunity between *Salmonella* and *E. coli*. There was a lack of evidence that anti-EcpA and -EcpD antibodies could bind to χ9558(pYA3337) based on Western blot and TEM. In addition, no reaction was observed when purified EcpA was probed with serum from χ9558(pYA3337). Previously, serum IgG cross-reactivity was demonstrated in RASV immunized mice against *E. coli* outer membrane proteins probably due to cross-reactivity of iron regulated outer membrane proteins (45), but to our knowledge, ECP or another homologous fimbria has not been reported in *Salmonella*. Anti-EcpA and -EcpD antibodies could be elicited by *Salmonella* antigens that share common conformational epitopes with ECP, which warrants future study.

Previously developed vaccines against ExPEC were tested mainly in BALB/c mice (36, 46–48); in this study, we evaluated our vaccines in both BALB/c and CBA/J mice. Slight differences in immune responses between mouse strains vaccinated with the same RASV could be due to a difference in *Salmonella* susceptibility. Contrary to BALB/c, CBA/J mice are considered more resistant to systemic infection with *S*. Typhimurium because these mice have a wild-type natural resistance-associated macrophage protein 1 allele conferring resistance to intracellular pathogens residing within vesicles (49). Furthermore, a higher median lethal dose is observed for CBA/J mice challenged with *S*. Typhimurium than BALB/c mice (50). However, a separate study showed both the spleen and liver are colonized by day 15 post-infection in CBA/J mice infected with *S*. Typhimurium strain 14028 (51). Because the parent *S*. Typhimurium strain χ3761 is more virulent than strain 14028 in mice (52), attenuated derivatives of χ3761 such as those used in this study should colonize internal organs as well, which explains elicitation of anti-*Salmonella* LPS IgG antibodies in the present study. LPS-specific antibodies were detected at 6 weeks but not 3 weeks postvaccination in BALB/c mice. These antibodies may have been elicited at 3 weeks postvaccination, but were not detected because they were in concentrations below the limit of detection in the ELISA. Previous studies have found no response or a low LPS-specific antibody titer in serum 2–3 weeks postvaccination with RASV strains (53, 54). However, by 6 weeks postvaccination LPS-specific antibodies were detected in these studies (53, 54). Mucosal IgA antibodies to LPS and ECP were also detected but only on day 41 in vaccinated BALB/c mice, which could not be compared to CBA/J mice at the same timepoint due to the UTI challenge timeline (Figure S2 in Supplementary Material).

Due to antigenic diversity of ExPEC strains, a vaccine should be designed to have broad protection. Previous studies have used O-antigen based vaccines against ExPEC serogroups commonly associated with human disease (5). One study used a tetravalent *E. coli* O-antigen vaccine to target serogroups O1, O2, O6, and O25 (55). Targeting specific O-groups based on prevalence in human patients is a rational approach, although it is limited to epidemiological evidence including region, as different countries may have different dominant serogroups (56). To assess protective potential of the RASV strains, *in vitro* assays were performed using multiple ExPEC serotypes and *S*. *enterica* serovars. Overall, mice vaccinated with χ9558(pYA4428) had the strongest inhibition of *E. coli* and *Salmonella* strains indicating inclusion of ECP may benefit broad protective ability. The mechanism of bacterial reduction in vaginal wash samples is unclear, but one proposed role of immunoglobulins in host defense includes antibodies binding to the bacterial surface and inhibiting its growth (57). Using an O1 specific monoclonal IgG antibody, Schauer et al. (58) found growth inhibition of *Cronobacter turicensis* after 2 h of incubation in an antibody concentration-dependent manner, independent of agglutination. A separate study found reduced growth of *E*. *coli* when incubated with IgY purified from egg yolk of White Leghorn hens immunized with formaldehyde-killed *E*. *coli* compared to IgY from unimmunized hens (59). RASVs eliciting elevated levels of anti-*E. coli* and *Salmonella* antibodies may have contributed to reduced bacterial levels in the *in vitro* assays. Other possibilities that may have accounted for differences in bacterial levels include transient differences in antimicrobial peptides in the samples collected, and reduced survival from complement-mediated effects instead of reduced bacterial growth. In addition, antibodies binding to pilus proteins may have caused agglutination of bacteria that was not detected in bacterial plating leading to an artificially low level in samples from immunized mice. Future studies could investigate the mechanisms responsible for the reduction in bacterial levels observed to determine whether these were biologically significant.

Recombinant attenuated *Salmonella* vaccines can be delivered orally which make them easy to administer to those at risk for ExPEC infection such as adults >50 years old, patients undergoing genitourinary surgery, and residents of long-term healthcare facilities (5). Concerns have been raised over oral vaccines negatively impacting the microbiome (60). However, recent studies have shown vaccination with a live attenuated *Salmonella* strain or conserved *E*. *coli* antigens had no influence on the intestinal microbiome (61, 62). Herein, the vaginal wash assay showed that vaccination with the RASV delivering ECP had a differential effect on commensal *E. coli*, which indicates vaccination could inhibit some commensal *E. coli* commonly found in the vaginal tract, but may be replaced by other strains that are not affected, such as the *E. coli* Nissle 1917.

Vaccines against human ExPEC-associated diseases have been tested in murine models to evaluate ability to protect against infection (36, 46–48). Previously, we found EcpA and EcpD recombinant antigens elicited protection in a lethal BALB/c mouse sepsis model (14). A separate study used a similar approach to the current strategy by selecting YncE as a vaccine antigen, which is highly conserved in *E. coli* (63). This study found reduction, but not elimination, of *E. coli* in the blood and organs of mice following intravenous challenge with CFT073. In the current study using a mouse sepsis model, vaccination with χ9558(pYA3337) but not χ9558(pYA4428) reduced bacterial loads in the spleen compared with unvaccinated mice, but no significant differences were found in the blood or liver between treatments. The reason for the inability of χ9558(pYA4428) to provide strong protection against CFT073 could be due to a lack of ECP synthesis in CFT073 during challenge. In previous studies, we found different protection levels in mice challenged with CFT073 grown in different conditions, and different growth conditions yielded different ECP profiles (10, 14). For χ9558(pYA3337) immunization, *Salmonella* LPS and core antigens may have elicited antibodies that are providing protection. Anti-core LPS antibodies raised in mice have been reported to bind both *E. coli* and *Salmonella* strains (64). Structurally, all *S. enterica* serotypes except Arizonae have identical LPS core structures, and for *E. coli* five different known core structures exist (65). Similarities between *Salmonella* and *E. coli* LPS cores and the elicitation of anti-LPS antibodies from vaccination could account for reduced loads in χ9558(pYA3337) vaccinated mice. In addition, antibody titer against LPS was slightly elevated in χ9558(pYA3337) mouse serum at day 41 compared with χ9558(pYA4428) and may account for protection seen with χ9558(pYA3337) vaccination. Although a significant difference was found in the spleen of χ9558(pYA3337) immunized mice, an early endpoint was used in the sepsis challenge, which may not translate to significant improvements in survival. This endpoint was used to determine bacterial loads in internal organs based on previous studies (35, 36). Future studies could assess whether reductions in bacterial loads correspond to significant improvements in survival.

In addition to the mouse sepsis model, CBA/J mice were used to assess vaccine protection against UTI. Both RASV strains were effective in reducing bacterial loads in the bladders of vaccinated mice. Inclusion of ECP was also significant when analyzing the number of *E. coli*-positive bladder and liver samples. Since IgA antibodies were not detected in immunized mice, this protection could be conferred by a systemic immunity type response. The IgG response was similar between RASV strains, and may account for why a similar level of protection was observed between RASV immunizations. Also, RASV strains carry pathogen-associated molecular patterns (e.g., flagella and LPS) that induce innate immunity (66). Recently, Powell et al. (67) showed that RASV strains, including those derived from χ3761, elicit distinct innate immune responses in mice. In our study, induction of innate immune cells likely played a role in reducing severity of sepsis and UTI in RASV immunized mice compared with unvaccinated mice.

Interestingly, the *in vitro* assays showed that challenge strain CFT073 was significantly reduced compared with the unvaccinated group in vaginal wash samples but not in sera from χ9558(pYA4428) immunized mice. This parallels the significant difference found in the mouse UTI model and the lack of significant differences observed in the sepsis challenge. Contrary to CFT073, we found reduced levels of other ExPEC strains, including the multidrug resistant ExPEC strain JJ1886 in bacterial inhibition assays. Additional ExPEC strains could be assessed in the mouse sepsis model in future studies to determine whether RASV immunization can provide stronger protection against different ExPEC, specifically those of sequence type 131 which are disseminated globally (5).

In summary, we found ECP proteins can be synthesized using a single plasmid as surface antigens on a RASV strain. Both RASV strains elicited an antibody response in mice, although it was detectable sooner with χ9558(pYA4428) for some antigens. The results suggest that the RASV alone or containing ECP showed potential of bacterial inhibition as assessed in *in vitro* assays and provided protection against *in vivo* UTI in the bladder. Future studies should optimize the ExPEC antigens displayed by the RASV strain for a stronger immune response and enhanced protection against ExPEC infection.

## Ethics Statement

This study was carried out in accordance with the recommendations of Arizona State University Institutional Animal Care and Use Committee. The protocol (#1168R) was approved by the Arizona State University Institutional Animal Care and Use Committee. Six-week-old female BALB/c mice (Charles River Laboratories, Wilmington, MA, USA) and 4-week-old female CBA/J mice (Jackson Laboratories, Bar Harbor, ME, USA) were obtained for infection experiments. Mice were acclimated for 7 days before experiments began. During the experiments, animals were monitored twice daily by our team, animal caretakers, and further inspected by a veterinarian.

## Author Contributions

Conceived and designed the experiments; performed the experiments: JM, ZS, and MM. Analyzed the data and reviewed and edited the manuscript: JM, ZS, RC, and MM. Contributed reagents/materials/analysis tools: RC and MM. Wrote the paper: ZS and MM.

## Conflict of Interest Statement

The authors declare that the research was conducted in the absence of any commercial or financial relationships that could be construed as a potential conflict of interest.
