# Supplementary material for: Evaluation of Recombinant Attenuated Salmonella Vaccine Strains for Broad Protection against Extraintestinal Pathogenic Escherichia coli
Source: Front Immunol. 2017 Oct 9;8:1280. doi: 10.3389/fimmu.2017.01280 (PMC5640888; doi:10.3389/fimmu.2017.01280)
Supplement: Supplementary file 1 [file data_sheet_1.docx]

***Supplementary Material***

**Evaluation of Recombinant Attenuated *Salmonella* Vaccine Strains for Broad Protection Against Extraintestinal Pathogenic *Escherichia coli***

**Jacob T. Maddux, Zachary R. Stromberg, Roy Curtiss III, Melha Mellata^*^**

#### ^*^Correspondence: Melha Mellata: [mmellata@iastate.edu](mailto:mmellata@iastate.edu)

**Supplementary Figures**

**
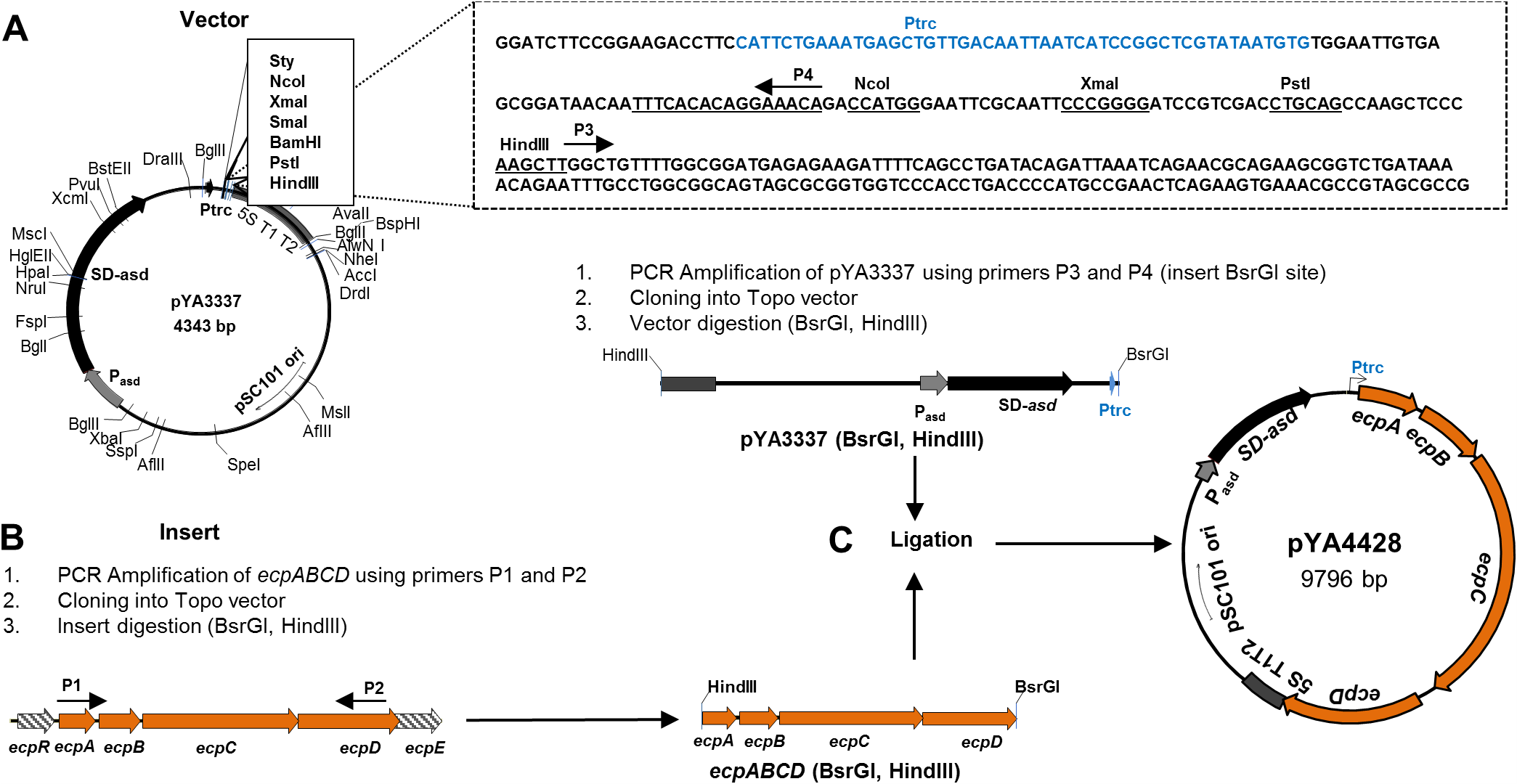
**

**Figure S1. Schematic of vector constructed for delivery of *Escherichia coli* antigens EcpA and EcpD.** (**A**) The map of the plasmid pYA3337 and Ptrc promotor sequence are shown. (**B**) The *ecp operon* was amplified from *E. coli*. (**C**) The BsrGI site was inserted into the *asd*-positive vector pYA3337 by PCR. The *ecpABCD* genes amplified from *E.* *coli* were cloned into the *asd*-positive vector with BsrGI to generate pYA4428.

**
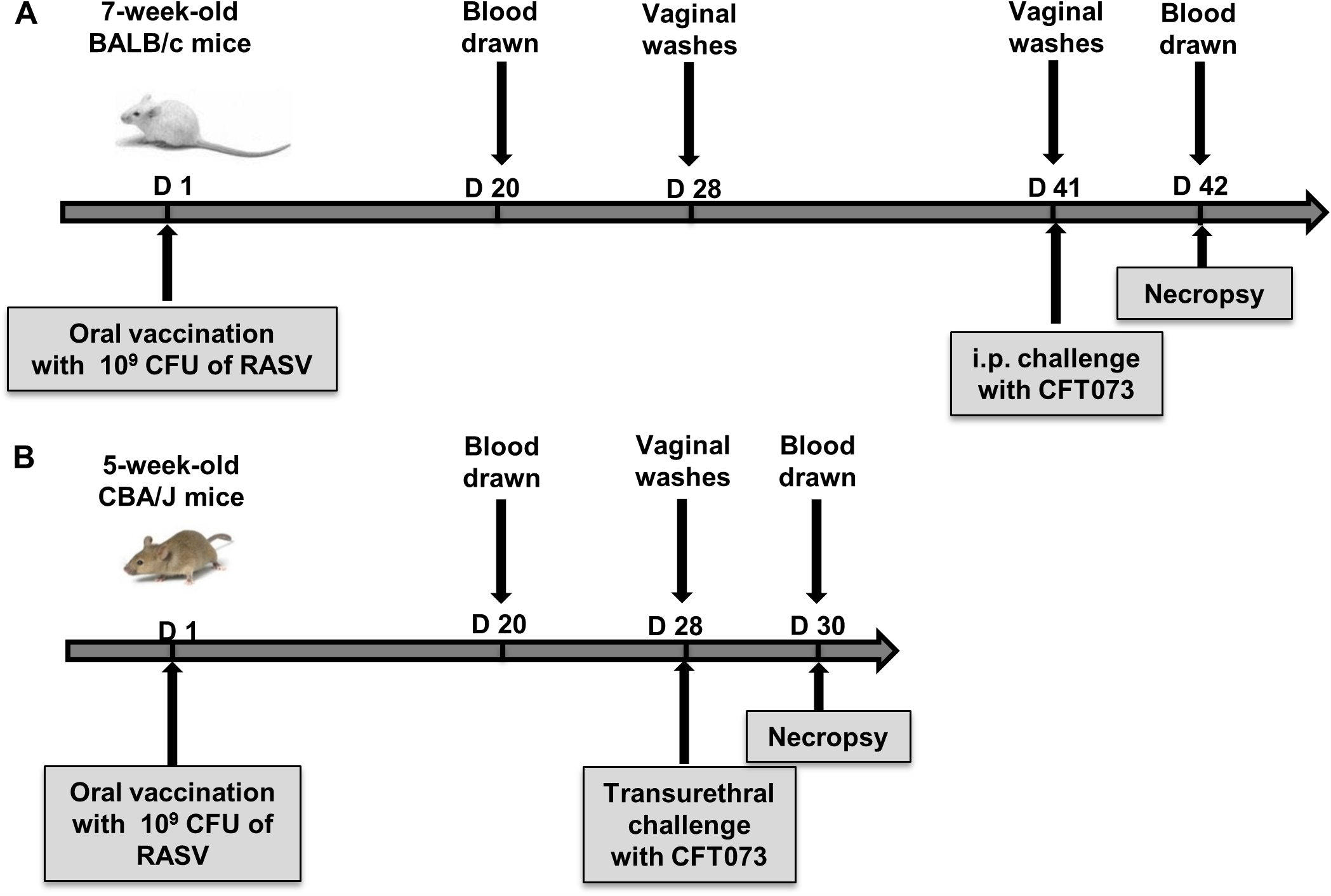
**

**Figure S2. Vaccination and challenge schedules.** (**A**) Seven-week-old BALB/c mice were vaccinated orally at day 1 and intraperitoneally (i.p.) challenged at day 41 to evaluate the ability of the vaccine treatment to protect against sepsis infection. (**B**) Five-week-old CBA/J mice were vaccinated orally at day 1 and transurethrally challenged at day 28 to evaluate the ability of the vaccine treatment to protect against urinary tract infection.


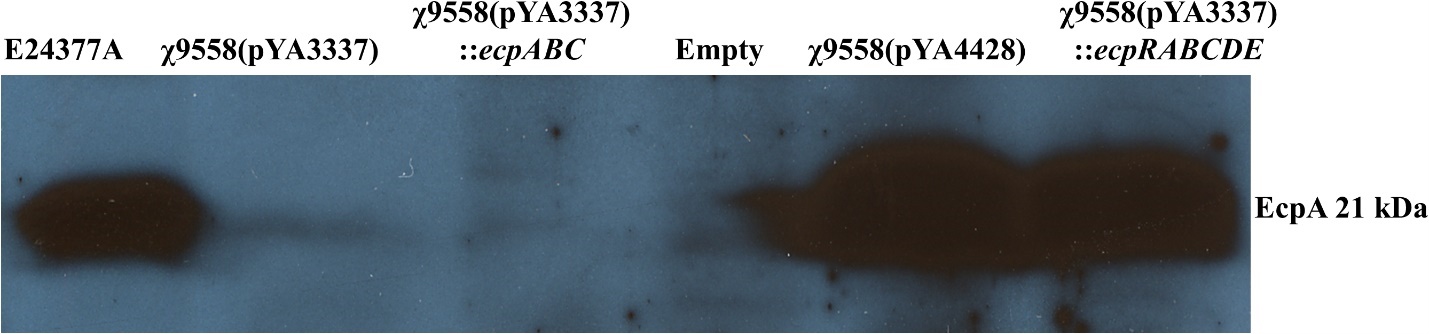


**Figure S3. *Escherichia coli* common pilus (ECP) synthesis in bacterial strains.** Western blot analysis showed ECP synthesis in positive control *E. coli* strain E24377A, attenuated *Salmonella* strain χ9558(pYA4428), and χ9558(pYA3337) carrying *ecpRABCDE*. χ9558 carrying only the empty plasmid pYA3337 or carrying *ecpABC* were deficient in ECP synthesis. The empty lane was not loaded.

**
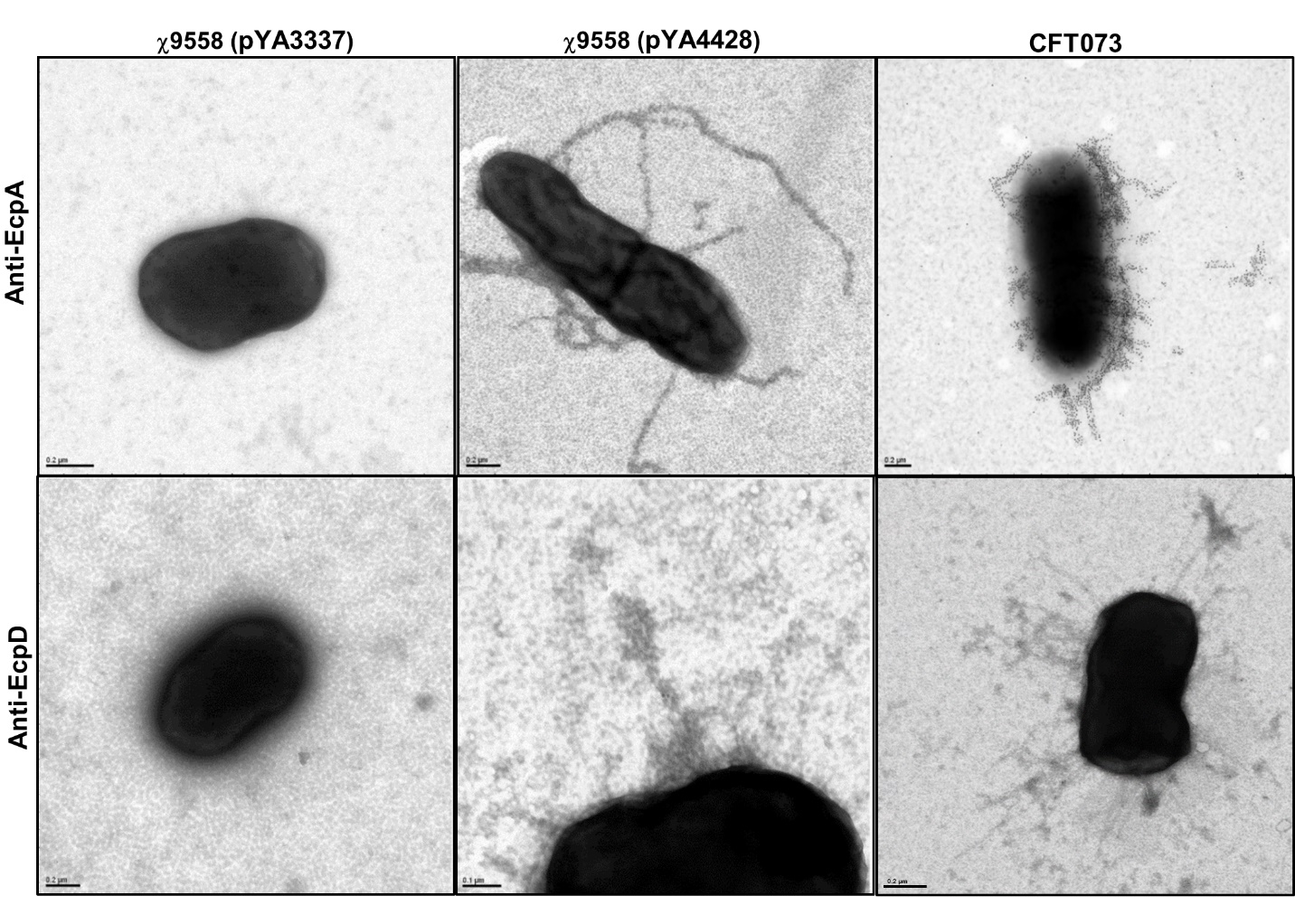
**

**Figure S4. *Escherichia coli* common pilus (ECP) expression on the surface of bacteria by immunoelectron microscopy.** EcpA and EcpD synthesis is shown in the *E. coli* wild-type strain CFT073 and RASV χ9558(pYA4428) containing the *asd*-plasmid with *ecpABCD*. The RASV χ9558(pYA3337) with an empty plasmid was deficient in EcpA and EcpD synthesis.


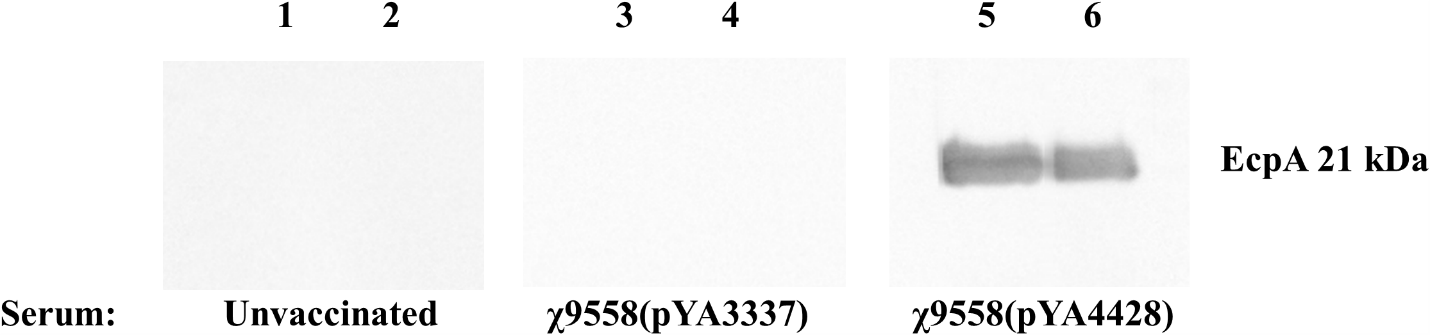


**Figure S5. Reaction of pooled serum samples from BALB/c mice against *Escherichia coli* common pilus (ECP).** All reactions were performed in duplicate using serum pooled in equal amounts from 10 mice. Western blot analysis showed no reaction to purified EcpA when probed with serum extracted from unvaccinated (lanes 1 and 2) and χ9558(pYA3337) (lanes 3 and 4) immunized mice. A positive reaction was observed for serum from χ9558(pYA4428) immunized mice (lanes 5 and 6).
